# Variation of the Bovidae (Mammalia: Artiodactyla) mitochondrial DNA control region and their phylogenetic relationship

**DOI:** 10.1080/23802359.2018.1492885

**Published:** 2018-07-31

**Authors:** Fang Zhao, Gonghua Lin, Zuhao Huang

**Affiliations:** a School of Life Sciences, Jinggangshan University, Ji’an, Jiangxi Province, China;; b Key Laboratory of Adaptation and Evolution of Plateau Biota, Northwest Institute of Plateau Biology, Chinese Academy of Sciences, Xining, China

**Keywords:** Mitochondrial DNA, control region, Bovidae, variation, phylogeny

## Abstract

The control region is the major noncoding segment of animal mitochondrial DNA. The mammal family Bovidae comprises all artiodactyl ungulates. To infer the organization and variation of Bovidae mitochondrial DNA control region, the complete control region sequences of 91 species were analysed. The control region ranged from 677 bp (*Saiga tatarica*) to 1329 bp (*Oryx dammah*) in length and can be separated into three domains among these species. The control region has the same flanking gene order from tRNA^pro^ to tRNA^Phe^. Genetic distances between species ranged from 0.44% (between *Bos javanicus* and *Bos taurus*) to 24.05% (between *Syncerus caffer* and *Gazella subgutturosa*). The average genetic distances among the species within the genera varied from 2.78% (*Procapra*) to 22.07% (*Hemitragus*). The average genetic distances showed significantly negative correlation with ts/tv. The maximum-likelihood method was used to construct a phylogenetic tree. Members of Bovinae appear in basal position among the Bovidae lineage. Analysis of control region genes supported the hypothesis of polyphyly for Antilopinae.

Mitochondrial genome has been a popular marker of choice for elucidating the phylogenetic relationships of recently diverged species as it is maternally inherited. The control region is the main regulatory region for transcription and replication of mtDNA. The mammal family Bovidae comprises all artiodactyl ungulates possessing nondeciduous horn cores and sheaths and includes antelopes, cattle, sheep, and goats (Gatesy et al. 1992). Although diverse taxa of mammals share the general structure (Pesole et al. 1999; Hassanin et al. 2009; An et al. 2010), the rates and patterns of molecular evolution of Bovidae control region are not known. In the present study, we examined the organization and variation of the control region of Bovidae species retrieved from GenBank. The aims are (1) to characterize the structural features and patterns of sequence evolution of the Bovidae mtDNA control region and (2) to infer the phylogeny of the Bovidae based on mtDNA control region.

A total of 91 species from 42 genera belonging to the Bovidae family was analysed. To ensure the entire mitochondrial DNA control region, only mitochondrial complete genome were selected. All the Bovidae species had only one control region. The control region spans the region between the genes for tRNA^Pro^ and tRNA^Phe^ in the Bovidae species, which was inconsistent with most of the avian species, between tRNA^Glu^ and tRNA^Phe^ (e.g. Huang and Ke 2017). The length of the control region sequences ranged from 677 bp (*Raphicerus campestris*) to 1329 bp (*Oryx dammah*), with an average size of 924 bp.

The average nucleotide composition of Bovidae control region sequences was as follows: 31.88%A, 28.15%T, 15.50%G, and 24.47%C, with a bias against G. The amount of A + T was more than that of G + C among whole control region. Bovidae control region sequences were alignable with certainty within genus. Genetic distances between species ranged from 0.44% (between *Bos javanicus* and *Bos taurus*) to 24.05% (between *Syncerus caffer* and *Gazella subgutturosa*), showing a wide range of divergences. The average genetic distances among the species within the genera varied from 2.78% (*Procapra*) to 22.07% (*Hemitragus*). The average genetic distances showed significantly negative correlation with ts/tv (*r* = −0.781, *p* < 0.01).

On the basis of hierarchical likelihood-ratio tests (hLRTs) as implemented in Modeltest 3.0, the model Hasegawa–Kishino–Yano (HKY) model + Gamma distribution + invariable sites was used (HKY + G + I, −lnL = 10551.06, *p* < 0.001, AIC = 21841.59, BIC = 23143.64). We set the shape of the Gamma distribution and the proportion of invariant sites as 0.32 and 0.09 (estimated by Modeltest), respectively. Maximum-likelihood phylogenetic tree (Figure 1) was estimated with the best-fit model HKY + G + I. All the species could be discriminated by their distinct clades in the phylogenetic tree (Figure 1). Members of Bovinae were the first to split from the Bovidae lineage. Analysis of control region genes supported the Bovidae fell into seven clades. Control region gene supported the subfamily Peleinae was sister-taxon to Reduncinae (Figure 1). Analysis of control region genes supported the hypothesis of polyphyly for Antilopinae.

**Figure 1.**
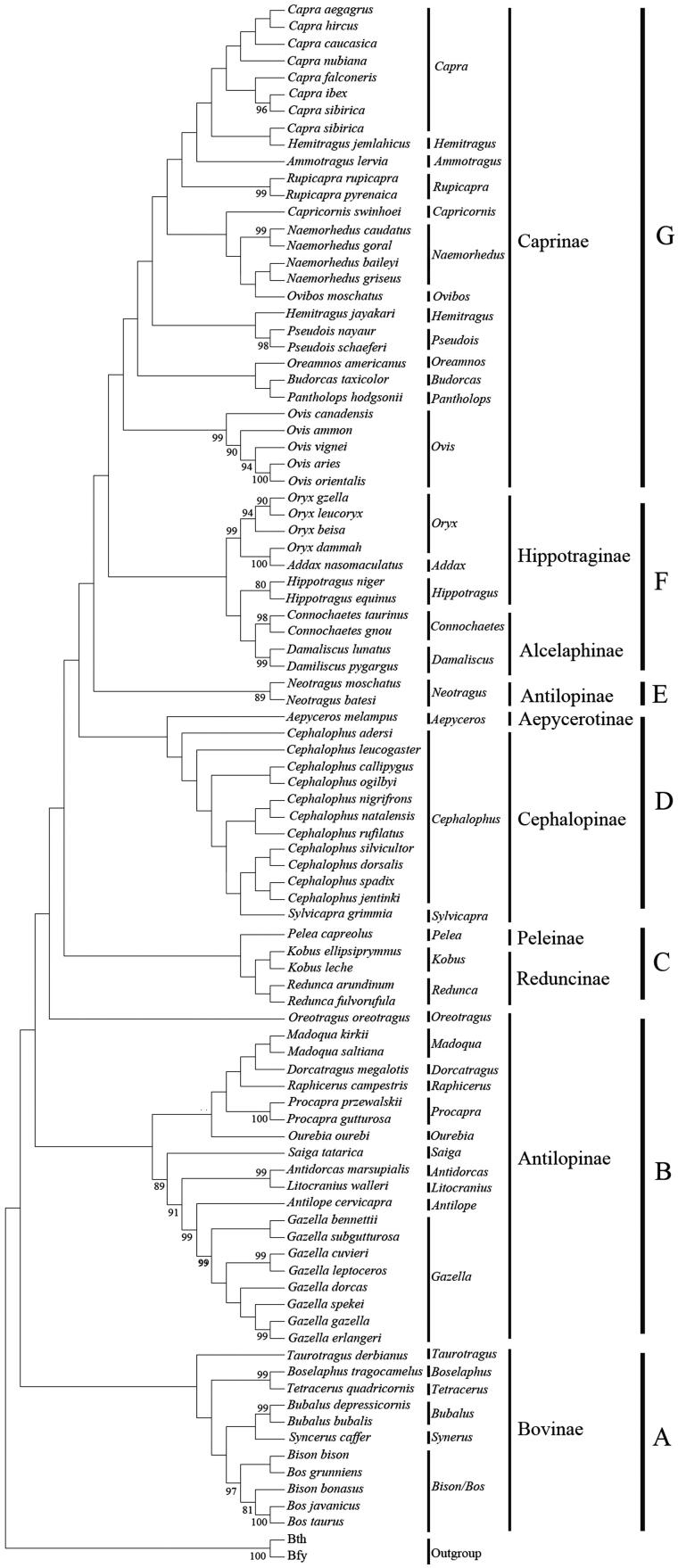
Maximum-likelihood tree of Bovidae constructed from control region genes. Numbers (in internodes) represent bootstrap values (>80%) from 1000 replications.
